# True metabolizable energy and amino acid digestibility in black soldier fly larvae meals, cricket meal, and mealworms using a precision-fed rooster assay

**DOI:** 10.1016/j.psj.2021.101146

**Published:** 2021-03-21

**Authors:** N. Matin, P. Utterback, C.M. Parsons

**Affiliations:** University of Illinois, Urbana, IL 61801, USA

**Keywords:** black soldier fly, cricket, mealworm, insect, poultry

## Abstract

Six precision-fed rooster assays were conducted to determine nutrient composition, nitrogen-corrected true metabolizable energy (**TME_n_**) and standardized amino acid digestibility for three black soldier fly larvae meals (**BSFL**), one partially-defatted BSFL, one cricket meal and two mealworm meals. The TME_n_ values were determined in three 48-h rooster assays using conventional roosters and the standardized amino acid digestibility values were determined in three 48-h rooster assays using cecectomized roosters. Nutrient analysis (DM basis) of the meals indicated that the CP varied from 45 to 58% among the four BSFL, was 67% for the cricket meal and varied from 51 to 56% for the two mealworms. Crude fat (12–30%), total P (0.7–1.1%), Ca (0.04–3.6%), and neutral detergent fiber (10–36%) also varied among the insect meals. The TME_n_ values for the three BSFL were generally consistent and averaged 4079 kcal/kg DM. As expected, partially-defatted BSFL contained a lower level of TME_n_. The TMEn of the cricket meal was 4223 kcal/kg DM. Due to their low fiber content and high fat content, the TME_n_ values for the two mealworms were high and in excess of 5000 kcal/kg DM. Amino acid concentrations of the various insect meals ranged from 0.69 to 1.1% for methionine, 0.57 to 0.73% for cystine, 3.3 to 4.5% for lysine, and 1.9 to 2.6% for threonine. Standardized amino acid digestibility values were generally high (most were 85–95%) for the four BSFL and two mealworms. Digestibility values for most amino acids were slightly lower for the cricket meal. Digestibility of cystine and valine were generally lower and more variable than other amino acids in the seven insect meals. The results of this study indicated that nutrient composition varies substantially among different insect meals, but all insect meals contained high levels of TME_n_ and digestible amino acids compared with feed ingredients commonly used in poultry diets.

## INTRODUCTION

The use of insect meals as a feed ingredient in poultry diets may be beneficial in poultry production. Increasing prices and depletion of conventional protein sources may result in a need to move towards more novel and non-traditional protein sources with similar nutritional potential. Insect meal has potential as a feed ingredient for poultry as it is high in protein, fat, minerals, and vitamins (Khusro et al., 2012). One of the most common types of insect meals, black soldier fly larvae (**BSFL**), contains 35–40% CP on a DM basis although this value can vary (Elwert et al., 2010). Other insect meals such as cricket meal and mealworms have been shown to contain 55–64% CP and 53% CP on a DM basis, respectively (Jones et al., 1972; Barker et al., 1998). Some of these protein values are similar to or exceed that of dehulled solvent-extracted soybean meal (**SBM**) and fish meal (NRC, 1994). With high percentages of protein being reported in the literature for various insects, the concentration and digestibility of each individual amino acid present is important to consider for determining the quality of the protein. The digestible amino acid values for poultry are also of importance to be able to better compare insect meals with conventional protein sources and to provide accurate values for use in feed formulation.

The precision-fed rooster assay is a common method used with conventional and cecectomized roosters to measure true metabolizable energy corrected to zero nitrogen retention (**TMEn**) and amino acid digestibility, respectively (Parsons et al., 1982; Engster et al., 1985). In the precision-fed assay, roosters are usually fasted for 24–26 h and then crop-intubated with approximately 30 g of a test ingredient, followed by being fasted for an additional 48 h during which time excreta are collected quantitatively. As mentioned above, the TME values are generally corrected to zero nitrogen balance (TME_n_) to prevent overestimation of metabolizable energy values (Parsons et al., 1982). Conventional roosters are used for determining TME_n_ because using cecectomized roosters would likely lead to underestimation of TME_n_ values since cecectomized roosters may not be able to derive as much energy from the diet as conventional roosters (Latshaw and Freeland, 2008). Conversely, cecectomized roosters are used for amino acid digestibility to obtain more accurate values (Parsons, 1986). The precision-fed rooster assay has provided a large amount of information on TME_n_ and amino acid digestibility for a variety of feedstuffs; however, it has been used only to a limited extent for insect meals. One precision-fed rooster assay done with field crickets as the test feedstuff determined the TME_n_ to be 2960 kcal/kg on an as-fed basis and the average true amino acid digestibility coefficients were found to be high and exceed those of fish meal (Wang et al., 2005). The objective of the current study was to use the precision-fed rooster assay to determine the amino acid digestibility and TME_n_ of several different kinds of insect meals including BSFL, crickets, and mealworms.

## MATERIALS AND METHODS

The protocol for this study was reviewed and approved by the Institutional Animal Care and Use Committee (Animal Use Protocol 20131)

### Ingredients and Analyses

There were a total of seven insect meals obtained from various companies for these studies. This included three samples of dried BSFL, one partially-defatted BSFL, one cricket meal, and two samples of mealworms. Analyses were conducted on these meals to determine N for crude protein (**CP**) via combustion (Method 990.03; AOAC International, 2007), crude fat (Method 920.93 A; AOAC International, 2007), acid detergent fiber (**ADF**) (Method 973.18;AOAC International, 2007), neutral detergent fiber (**NDF**) (Method 2002.04; AOAC International, 2007), and mineral concentrations of Ca, P, and Na by inductively coupled plasma optical emission spectroscopy (Method 985.01 A, B, and D; AOAC International, 2007). Amino acid concentrations were additionally analyzed (Method 982.30 E [a, b, and c]; AOAC International, 2007) with methionine and cystine being analyzed as methionine sulfone and cysteic acid after performic acid oxidation. All of the above analyses were conducted at the University of Missouri-Columbia Experiment Station Chemical Laboratory. Gross energy (**GE**) was analyzed using a bomb calorimeter (Model 6300; Parr Instruments, Moline, IL) and dry matter (**DM**) content was determined (Method 930.15; AOAC International, 2007), both at the University of Illinois.

### Diets and Design

Six experiments were conducted with Single Comb White Leghorn roosters using the precision-fed rooster assay (Parsons et al., 1982). The only aspects of experimental design varying among experiments were the kind/amount of sample fed and the sample size.

Experiment 1 was conducted to determine TME_n_ of BSFL 1 and BSFL 2 using conventional roosters. These BSFL samples were obtained from different sources where BSFL 1 were harvested between 10 and 14 d of age and BSFL 2 were harvested just prior to when they start to darken into prepupae which on average will be within 3 wk. The roosters were fasted for 26 h and then precision-fed 25-30g of the two BSFL. There were eight replicates of one individually-caged rooster per each treatment. For 48-h post-feeding, excreta were quantitatively collected on trays placed under each rooster and then excreta samples were immediately placed in a freezer. These excreta samples were later freeze-dried, weighed, ground, and analyzed for GE and N as described above. The TME_n_ values were then calculated as described by Parsons et al. (1982).

Experiment 2 was similar to Experiment 1 except that standardized amino acid digestibility of the BSFL 1 and BSFL 2 was determined using cecectomized roosters. The procedures and number of birds per sample were the same as Experiment 1. The excreta collected from the birds in this experiment were analyzed for amino acids as described above. Standardized amino acid digestibility values were calculated using the amino acid excretion of fasted roosters for the basal endogenous amino acid correction (Parsons, 1986).

Experiment 3 followed the same procedures as Experiment 1 and was conducted to determine the TME_n_ of BSFL 3 and a cricket meal. There were six conventional roosters per treatment.

Experiment 4 followed the same procedure as Experiment 2 and was conducted to determine standardized amino acid digestibility of BSFL 3 and a cricket meal. The BSFL 3 was harvested between 18 and 20 d post-hatch. There were six cecectomized roosters per treatment.

Experiment 5 was another rooster assay conducted to determine TME_n_ of one partially-defatted BSFL and two different mealworms (mealworm 1 and 2). There were six conventional roosters per treatment.

Experiment 6 was the final rooster assay and was conducted to determine standardized amino acid digestibility of the partially-defatted BSFL and the two mealworms. There were six cecectomized roosters per treatment.

Excreta collected for TME_n_ in Experiments 3 and 5 were analyzed for GE and N using the same methods as for Experiment 1 and TME_n_ values were also calculated by the same method. Excreta collected for amino acid digestibility in Experiments 4 and 6 were analyzed for amino acids using the same methods as for Experiment 2. Standardized amino acid digestibility values were also calculated by the same method as in Experiment 1.

### Statistical Analysis

The SAS software (SAS institute. INC., 2010) was used to statistically analyze the data obtained from each of the six experiments. An ANOVA was initially completed within the software for completely randomized designs. The least significant difference test was then conducted to provide information on whether the differences between or among treatments were significant at *P* < 0.05. For all statistical analyses, the experimental unit was the individual rooster.

## RESULTS AND DISCUSSION

### Chemical Analysis

The analyzed nutrient compositions of BSFL 1 and BSFL 2 (Experiment 1 and 2), BSFL 3 and cricket meal (Experiment 3 and 4), and partially-defatted BSFL meal, Mealworm 1 and 2, (Experiment 5 and 6) are shown in Table 1, Table 2, Table 3. The nutrient composition of BSFL 1 and 2 were generally similar to one another (Table 1). Both were high in CP (approximately 58%) and contained approximately 20% fat. The CP of BSFL 3 (Table 2) was somewhat lower than BSFL 1, 2, and the partially-defatted BSFL (Table 3), whereas the NDF and Ca levels were much higher in BSFL 3. The Ca levels for the four BSFL (Table 1, Table 2, Table 3) were considerably lower than the Ca level of defatted BSFL meal reported earlier by Marono et al. (2017) to be 7.06% on a DM basis. The Ca level of the partially-defatted BSFL in the current study was, however, in close agreement with the Ca level of 1.02% on a DM basis as analyzed for partially-defatted BSFL meal by Maurer et al. (2016).

**Table 1:** Analyzed composition and TME_n_ of two black soldier fly larvae meals (BSFL) (DM basis).^1^

	BSFL 1	BSFL 2	SEM
Crude protein (%)	57.5	58.1	
Crude fat (%)	20.7	19.2	
Acid detergent fiber (%)	7.44	9.85	
Neutral detergent fiber (%)	12.4	15.3	
Ca (%)	1.34	2.28	
P (%)	1.03	1.11	
Na (%)	0.12	0.17	
Gross energy (kcal/kg)	5410	5325	
TME_n_ (kcal/kg)	4250^a^	3998^b^	0.053

^a-b^Values with different superscripts are significantly different (*P* < 0.05).

1 TME_n_ values are means of 8 individually-caged conventional roosters. All other values are means of duplicate analyses.

**Table 2:** Analyzed composition and TME_n_ of black soldier fly larvae meal (BSFL) and cricket meal (DM basis).^1^

	BSFL 3	Cricket	SEM
Crude protein (%)	45.2	67.4	
Crude fat (%)	19.0	19.4	
Acid detergent fiber (%)	8.17	13.1	
Neutral detergent fiber (%)	32.7	36.0	
Ca (%)	3.65	0.14	
P (%)	0.85	0.82	
Na (%)	0.18	0.43	
Gross energy (kcal/kg)	5159	5877	
TME_n_ (kcal/kg)	3990^b^	4223^a^	0.065

^a-b^Values with different superscripts are significantly different (*P* < 0.05).

1 TME_n_ values are means of 6 individually-caged conventional roosters. All other vales are means of duplicate analyses.

**Table 3:** Analyzed composition and TME_n_ of partially-defatted black soldier fly larvae meal (BSFL) and two mealworm meals (DM basis).^1^

	Partially-defatted BSFL	Mealworm 1	Mealworm 2	SEM
Crude protein (%)	56.7	50.9	55.8	
Crude fat (%)	12.2	29.8	30.4	
Acid detergent fiber (%)	13.8	6.25	7.21	
Neutral detergent fiber (%)	21.2	9.51	13.1	
Ca (%)	0.91	0.04	0.04	
P (%)	0.87	0.76	0.71	
Na (%)	0.24	0.16	0.10	
Gross energy (kcal/kg)	5110	6144	6189	
TME_n_ (kcal/kg)	3561^c^	5125^b^	5273^a^	0.038

^a-c^Values with different superscripts are significantly different (*P* < 0.05).

1 TME_n_ values are means of 6 individually-caged conventional roosters. All other values are means of duplicate analyses.

The total P, Na, and GE levels did not vary greatly among the three BSFL (Tables 1 and 2). The total P of the three BSFL and partially-defatted BSFL were similar to previously reported BSFL which contained approximately 0.90% P on a DM basis (Newton et al., 1977; Arango Gutierrez et al., 2004; Makkar et al., 2014). The CP of the partially-defatted BSFL was similar to that of BSFL 1 and 2 but was higher than BSFL 3. Previous studies suggested that defatting BSFL will increase the CP concentration of the meal (Makkar et al., 2014; Spranghers et al., 2017; Mwaniki and Kiarie, 2018); however, this was not observed consistently for BSFL obtained from different sources in the present study. Possible explanations for this could be that the insects for BSFL 1 and BSFL 2 were reared on diets containing higher CP concentrations compared with the insects for partially-defatted BSFL and that the partially- defatted BSFL contained a higher level of acid detergent fiber and neutral detergent fiber which diluted the CP. As expected, the fat level and GE level in the partially-defatted BSFL were lower than the BSFL 1-3. Similar results were obtained when Schiavone et al. (2017) compared BSFL with 18% fat (DM basis) and BSFL with 4.6% fat (DM basis) such that the lower fat BSFL had a lower GE compared with the BSFL containing a higher fat concentration. The ADF and Na concentration in the partially-defatted BSFL were higher than for the three BSFL, whereas level of NDF, Ca, and P were within the range observed for the three BSFL. The CP of all the BSFL are either higher than or similar to the CP value of 48% reported in the NRC (1994) for dehulled SBM.

The cricket meal was higher in CP, fiber, and Na than the three BSFL. A higher Na value was expected for cricket meal compared with the other insect meals as crickets have a high dietary requirement for Na (Nakagaki et al., 1987). Irungu et al. (2018) analyzed the mineral composition of adult cricket meal and obtained a much higher mean Na value of 2.26% (DM basis) compared with the present cricket meal, whereas Nakagaki et al. (1987) obtained a more similar value of 0.92% Na (DM basis). The 64% CP for our cricket meal was in general agreement with the concentration of approximately 60–62% CP in crickets reported by Nakagaki et al. (1987) and Finke (2015). The level of fat in our cricket meal was higher than the cricket meal evaluated by Finke (2015).

The composition of the two mealworms was generally similar to one another (Table 3). Both were higher in fat than the BSFL and cricket meals and were generally lower in NDF. For Ca, mealworms also had the lowest Ca values compared to all the other insect meals. The latter was expected as mealworms are poor sources of Ca and Ravzanaadii et al. (2012) and Finke (2015) also determined mealworm larvae to have a low Ca content of 0.04–0.05% which agreed well with the present findings. When comparing the Ca content across all insect meals evaluated herein, all BSFL meals had higher levels of Ca compared with cricket meals and both mealworm meals. Calcium is stored and converted to CaCO_3_ within the BSFL and is the mineral present in the highest abundance within their bodies (Tomberlin et al., 2002). This level of Ca has been shown to be over ten times higher than the amount of Ca present in most other insects (Wang and Shelomi, 2017). The variability in Ca and other nutrients analyzed in BSFL meal samples can probably be mainly attributed to the variability in the nutritional composition of the diet that was fed to the larvae (Newton et al., 2008).

## TMEn AND STANDARDIZED AMINO ACID DIGESTIBILITY

### Experiments 1 and 2

The TME_n_ value for BSFL 1 was significantly higher than that of BSFL 2 (Table 1). A possible explanation for this is that the higher fiber and mineral content observed in BSFL 2 could have contributed to its lower TME_n_ value. Each of the TME_n_ values of 4250 and 3998 kcal/kg DM are higher than the NRC (1994) reported value of 2761 kcal/kg DM for dehulled solvent extracted SBM.

In regards to standardized digestibility of amino acids, BSFL 1 had significantly higher digestibility values for threonine, valine, methionine, isoleucine, leucine, arginine, tyrosine, aspartate, serine, and phenylalanine compared with BSFL 2 (Table 4). Digestibility of other amino acids did not significantly differ between these meals. The digestibility of most of the amino acids in BSFL 1 and 2 were similar to or slightly higher than NRC (1994) values for SBM. Two notable exceptions were that the digestibilities of cystine and valine were somewhat lower than NRC (1994) values for SBM. Comparing the standardized amino acid digestibility values from cecectomized roosters fed BSFL 1 and 2 with a study by Mwaniki and Kiarie (2018) that measured standardized ileal digestibility (**SID**) in broiler chicks fed BSFL, the latter study generally reported slightly lower amino acid digestibility values. For example, SID values obtained by Mwaniki and Kiarie (2018) were 86.3% and 88.7% for lysine and methionine, respectively.

**Table 4:** Total amino acids, standardized amino acid digestibility values, and digestible amino acid concentrations for two black soldier fly larvae meals (BSFL) (%) (DM basis).

Amino Acid	Total	BSFL 1 Digest. value	Digest. conc.^1^	Total	BSFL 2 Digest. value	Digest. conc.^1^	SEM^2^
ASP	5.22	92.90^a^	4.85	4.99	90.35^b^	4.51	0.49
THR	2.24	91.86^a^	2.06	2.24	88.11^b^	1.97	0.80
SER	2.03	90.57^a^	1.84	2.12	87.20^b^	1.85	0.96
GLU	5.65	91.25^a^	5.16	5.82	89.25^a^	5.19	0.75
PRO	2.72	91.58^a^	2.49	3.09	89.65^a^	2.77	0.86
GLY	3.02	-	-	3.47	-	-	-
ALA	3.66	92.32^a^	3.38	4.16	91.43^a^	3.80	0.48
CYS	0.60	80.32^a^	0.48	0.67	73.36^a^	0.49	2.42
VAL	4.16	81.23^a^	3.38	4.69	76.52^b^	3.59	1.06
MET	1.02	94.65^a^	0.97	0.95	92.48^b^	0.88	0.40
ILE	2.66	92.27^a^	2.45	2.56	90.41^b^	2.31	0.46
LEU	3.91	92.63^a^	3.62	3.82	90.54^b^	3.46	0.55
TYR	3.44	93.99^a^	3.23	3.49	91.13^b^	3.18	0.57
PHE	2.57	92.74^a^	2.38	2.43	90.67^b^	2.20	0.58
LYS	4.26	90.43^a^	3.85	4.48	88.17^a^	3.95	0.99
HIS	1.60	90.27^a^	1.44	1.95	88.62^a^	1.73	0.79
ARG	2.68	94.78^a^	2.54	2.86	92.70^b^	2.65	0.65
TRP	0.84	96.90^a^	0.81	0.83	97.20^a^	0.81	0.35

^a-b^Standardized digestibility values within the same row with different superscripts are significantly different (*P* < 0.05). Values are means of 8 individually caged cecectomized roosters.

1 Digestible concentration = (total x standardized digestibility values)/100.

2 SEM for standardized digestibility values.

### Experiments 3 and 4

These two experiments determined the TME_n_ (Table 2) and amino acid digestibility (Table 5) values for BSFL 3 and a cricket meal sample. The TME_n_ of the cricket meal was significantly higher than that of BSFL 3. Both BSFL 3 and cricket meal exceeded SBM (NRC, 1994) in regards to TME_n_. Except for cystine and lysine, all of the amino acid digestibility values for BSFL 3 were significantly higher than those for cricket meal. Arginine and methionine digestibility values of BSFL 3 are similar to values reported by the NRC (1994) for SBM. The values reported by Mwaniki and Kiarie (2018) for SID of broilers fed BSFL for lysine, cystine, valine, and arginine were higher than the current standardized amino acid digestibility values of BSFL 3 using cecectomized roosters. Conversely, their broiler SID values for methionine and threonine were lower than our rooster values for BSFL 3. In summary, our results for the three BSFL indicate that the amino acids are well digested by poultry which agrees well with the results by Hall et. al. (2018) who reported similar results in broilers for house fly larvae.

**Table 5:** Total amino acids, standardized amino acid digestibility values, and digestible amino acid concentrations for black soldier fly larvae meal (BSFL) and cricket meal (%) (DM basis).

Amino Acid	Total	BSFL 3 Digest. value	Digest. Conc.^1^	Total	Cricket Digest. value	Digest. Conc.^1^	SEM^2^
ASP	4.35	87.50^a^	3.81	6.81	78.46^b^		
	5.34	1.04					
THR	1.89	86.62^a^	1.64	2.63	84.18^a^	2.21	1.01
SER	1.85	86.40^a^	1.60	3.22	78.92^b^	2.54	1.45
GLU	5.40	87.66^a^	4.73	7.46	84.06^b^	6.27	0.71
PRO	2.96	88.52^a^	2.62	4.43	75.22^b^	3.33	0.95
GLY	3.27	-	-	6.90	-	-	-
ALA	3.23	89.37^a^	2.89	7.45	73.44^b^	5.47	0.57
CYS	0.57	66.49^a^	0.38	0.73	68.60^a^	0.50	3.74
VAL	4.08	76.38^a^	3.12	6.31	69.62^b^	4.39	1.92
MET	0.69	90.38^a^	0.62	1.13	88.47^b^	1.00	0.40
ILE	2.14	89.74^a^	1.92	3.31	82.50^b^	2.73	0.57
LEU	3.27	90.44^a^	2.96	5.39	83.66^b^	4.51	0.66
TYR	2.80	92.22^a^	2.58	3.69	77.20^b^	2.85	0.62
PHE	1.98	90.49^a^	1.79	2.54	85.71^b^	2.18	0.78
LYS	3.36	79.15^a^	2.66	4.32	80.62^a^	3.48	1.32
HIS	1.45	84.40^a^	1.22	1.77	77.59^b^	1.37	1.02
ARG	2.16	91.49^a^	1.98	4.17	88.27^b^	3.68	0.72
TRP	0.66	94.85^a^	0.63	0.70	92.67^b^	0.65	0.58

^a-b^Standardized digestibility values within the same row with different superscripts are significantly different (P<0.05). Values are means of 6 individually caged cecectomized roosters.

1 Digestible concentration = (total x standardized digestibility values)/100.

2 SEM for standardized digestibility values.

The cricket meal in the current study had amino acid concentrations generally similar to those of crickets reared on a dairy cow diet consisting mostly of cereals (Collavo et al., 2005). Two exceptions were large differences between studies for cystine and valine concentrations. Standardized amino acid digestibility of the cricket meal in the current study was lower for several amino acids of practical importance to poultry (such as threonine, cystine, valine, and lysine) when compared with SBM (NRC, 1994).

### Experiments 5 and 6

When comparing the two mealworm samples, Mealworm 2 was shown to have a significantly higher TME_n_ compared to Mealworm 1 (Table 3). As expected, the TME_n_ of partially-defatted BSFL meal was significantly lower than both mealworms due to its much lower fat content. The TMEn of partially-defatted BSFL and was also lower than the BSFL 1 and 2, again, probably due mainly to its lower fat content. The total concentration of each amino acid in the two mealworms was similar (*P* > 0.05). Total amino acid concentration values for these mealworms were in general agreement with those of De Marco et al. (2015) except for valine and cysteine. The 2.82% valine value obtained by De Marco et al. (2015) was approximately two times less than the valine value determined in the Mealworms 1 and 2 in the current study. Further studies conducted by Ravzanaadii et al. (2012) obtained a valine concentration of 2.44% on a DM basis, in close agreement with De Marco et al. (2015), while Bovera et al. (2015) obtained a higher valine value of 3.72% on a DM basis. All of these valine concentrations are lower than the mean value of 5.6% in the current study. In contrast to that lower level of valine, DeMarco et al. (2015) reported a higher cystine concentration of 1.25% in mealworms which is twice the concentration of cysteine observed in the current study. Thus, it seems that the amino acid levels in mealworms can vary among samples, at least for a few amino acids.

For the standardized amino acid digestibility of these three insect meals (Table 6), the mealworms did not significantly differ from one another for any amino acids. Both of these mealworms had significantly higher standardized amino acid digestibility values when compared with partially-defatted BSFL meal with the exception of cystine, methionine, and lysine which did not significantly differ among the insect meals. De Marco et. al. (2015) also reported high amino acid digestibility values for mealworms, and Biasato et. al. (2018) observed that feeding increased levels of the mealworms to broilers increased weight gain and feed intake but decreased feed efficiency. Although the methionine digestibility value of the partially de-fatted BSFL meal was similar to that of dehulled SBM in NRC (1994), the digestibility values of other amino acids, including cystine, lysine, threonine, and arginine, were all comparatively lower.

**Table 6:** Total amino acids, standardized amino acid digestibility values, and digestible amino acid concentrations for partially defatted black soldier fly larvae (BSFL) meal and two mealworms (%) (DM basis).

		Partially defatted BSFL			Mealworm 1			Mealworm 2		
Amino Acid	Total	Digest. value	Digest. conc.^1^	Total	Digest. value	Digest. conc.^1^	Total	Digest. value	Digest. Conc.^1^	SEM^2^
ASP	5.20	87.85^b^	4.57	4.39	93.10^a^	4.09	4.80	90.71^a^	4.35	0.80
THR	2.33	84.38^b^	1.97	2.19	92.05^a^	2.02	2.29	90.66^a^	2.08	1.11
SER	2.46	81.73^b^	2.01	2.36	89.77^a^	2.12	2.44	89.62^a^	2.19	1.35
GLU	6.47	86.58^b^	5.60	5.89	93.27^a^	5.49	6.43	92.57^a^	5.95	0.82
PRO	3.97	82.56^b^	3.28	3.80	90.97^a^	3.46	3.57	90.64^a^	3.24	1.06
GLY	4.44	-	3.00	3.25	-	-	3.29	-	-	-
ALA	3.88	83.95^b^	3.26	4.07	93.15^a^	3.79	4.06	92.04^a^	3.74	0.82
CYS	0.64	68.70^a^	0.44	0.69	75.79^a^	0.52	0.59	76.11^a^	0.45	3.62
VAL	7.45	59.72^b^	4.45	5.64	72.73^a^	4.10	5.74	73.90^a^	4.24	1.99
MET	0.92	91.68^a^	0.84	0.73	92.13^a^	0.67	0.78	90.99^a^	0.71	0.91
ILE	2.65	86.37^b^	2.29	2.56	92.06^a^	2.36	2.63	91.32^a^	2.40	0.89
LEU	4.07	85.78^b^	3.49	4.00	93.09^a^	3.72	4.18	92.41^a^	3.86	0.97
TYR	3.62	88.33^b^	3.20	3.58	92.61^a^	3.32	4.41	93.39^a^	4.12	0.77
PHE	2.46	88.38^b^	2.17	2.17	91.68^a^	1.99	2.35	91.21^ab^	2.14	1.00
LYS	3.88	88.50^a^	3.43	3.28	90.92^a^	2.98	3.54	88.72^a^	3.14	1.10
HIS	1.87	84.64^b^	1.58	1.66	91.39^a^	1.52	1.84	91.06^a^	1.68	0.94
ARG	2.84	90.77^b^	2.58	2.91	94.64^a^	2.75	2.95	93.98^a^	2.77	0.88
TRP	0.81	97.35^b^	0.79	0.61	100.20^a^	0.61	0.63	99.13^a^	0.62	0.42

^a-b^Standardized digestibility values within the same row with no common superscripts are significantly different (P<0.05). Values are means of 6 individually caged cecectomized roosters.

1 Digestible concentration = (total x standardized digestibility values)/100.

2 SEM for standardized digestibility values.

The standardized amino acid digestibility of valine in the partially-defatted BSFL meal was lower at 60% than other amino acids and was also lower compared with other BSFL samples evaluated in the earlier experiments of the current study. The digestible valine content of the partially defatted BSFL meal (∼60%) was the lowest of all of the amino acids in that sample, as well as in the other three BSFL samples. Taken together, the low valine digestibility may be common among different types of insect meals and may not be unique to BSFL. The low digestibility of valine may at least partially explain why, in a study evaluating defatted BSFL in laying hen diets (Mwaniki et. al, 2020), it was reported that increasing levels of the BSFL resulted in decreased egg mass and increased feed conversion ratio.

In summary, the results obtained from these experiments indicated that there is a high degree of variability in the nutrient composition among different insect meals. Cricket meal contained the highest percentage of crude protein and Na among all the insect meals analyzed. As expected, the BSFL samples contained the highest Ca values among all the insect meals. Mealworms had the highest TME_n_ values due to their high fat content although all of the insect meals were high in energy and contained TME_n_ values of 3561-5273 kcal/kg DM. High amino acid digestibility values were also generally observed among all of the insect meals. For most of the insect meals, cystine generally had the lowest digestibility while tryptophan had the highest digestibility in comparison with the other amino acids analyzed. Valine digestibility values were also generally low among the insect meals and ranged between 60–81%; cricket meal and the partially-defatted BSFL had the lowest values at 70% and 60%, respectively. An explanation for the low valine digestibility of insect meals is lacking and represents an area for further study. Cricket meal generally had a lower amino acid digestibility in comparison with the other insect meals. The latter may be at least partially associated with chitin content because chitin present in the insect meals binds to some of the protein within the insect, thereby making it indigestible (Rumpold and Schluter, 2015). Furthermore, Rumpold and Schlüter (2015) found that the removal of chitin in bees increased the percentage of digestible protein from 71.5 to 94.3%. Since the cricket meal in this experiment was harvested from adult insects, this could possibly partially explain the generally lower amino acid digestibility values compared with other insect meals. In conclusion, the results of the current study suggested that insect meals are a potential alternative feed ingredient for poultry production, as the metabolizable energy content and amino acid digestibility were similar to or higher than most conventional high-protein feed ingredients commonly used in poultry diets such as dehulled SBM.

## DISCLOSURES

The authors declare no conflicts of interest.
